# Does Current Knowledge Give a Variety of Possibilities for the Stabilization/Solidification of Soil Contaminated with Heavy Metals?—A Review

**DOI:** 10.3390/ma15238491

**Published:** 2022-11-28

**Authors:** Agnieszka Lal, Joanna Fronczyk

**Affiliations:** 1 Faculty of Civil Engineering and Architecture, Lublin University of Technology, 40 Nadbystrzycka Str., 20-618 Lublin, Poland; 2 Institute of Civil Engineering, Warsaw University of Life Sciences—SGGW, 166 Nowoursynowska Str., 02-787 Warsaw, Poland

**Keywords:** binder, amendments, additives, sample preparation, unconfined compressive strength—UCS, leachability tests

## Abstract

Stabilization/solidification of contaminated soil is a process that allows simultaneous strengthening of the soil structure, disposal of contamination and recycling of industrial waste, implemented as substitutes for Portland cement or additives to improve the properties of the final product obtained. Extremely intensive development of studies pertaining to the S/S process prompted the authors to systematize the binders used and the corresponding methods of binding the contamination, and to perform an analysis of the effectiveness expressed in geomechanical properties and leachability. The study pays close attention to the types of additives and binders of waste origin, as well as the ecological and economic benefits of their use. The methods of preparing and caring for the specimens were reviewed, in addition to the methods of testing the effectiveness of the S/S process, including the influence of aging factors on long-term properties. The results of the analyses carried out are presented in the form of diagrams and charts, facilitating individual evaluation of the various solutions for the stabilization/solidification of soils contaminated with heavy metals.

## 1. Introduction

In-situ stabilization/solidification of soils contaminated with heavy metals is the subject of worldwide research in the fields of chemistry, geotechnics and environmental engineering. It was originally a process used under the name of stabilization, only as an economical alternative to soil replacement, in case of insufficient strength and deformation properties of the subsoil [1,2,3,4,5,6,7,8,9,10]. However, due to its effective binding of contaminants at relatively low cost [11,12,13] and competitive time-consumption [14,15], it has become known as a leading method of soil remediation [16,17,18,19]. It is understood both as a natural subsoil, subjected to incidental contamination, and is still used for engineering tasks (including the foundation of cubic and linear structures), as well as subsoil, which is a substructure or another layer of a landfill, in which the presence of contaminants is long-lasting and intensified. There is a number of binders available for use in the stabilization/solidification process, the choice of which govern the method of minimizing heavy metal activity in the soil [20]. The neutralization of contaminants can take place only mechanically (due to the binding of pozzolanic materials), chemically (due to the reaction of contaminants with suitable compounds, resulting in the precipitation of metals in a less harmful form), by oxidation, chemical reduction or as a combination of the above [21,22,23]. Systematization of the variety and multiplicity of binders that are able to implement the S/S process is required. There have been attempts to create such a database [23,24,25,26]; however, due to the intensive development of research, there are deficiencies in the reported data. Waste materials with pozzolanic properties should have a special place in this type of statement. Their importance is highlighted due to the fact that they are perfectly in line with the sustainable construction trends being promoted. Nevertheless, it is also important to define the main types of tests that allow verification of the quality of the stabilization/solidification, expressed in the mechanical properties of the subsoil and the effectiveness in immobilization of contaminants. It is also necessary to gather information on the procedures of preparing samples for testing, as well as basic parameters and methods of curing. The aforementioned elements were analyzed on the basis of the studies available worldwide, mainly in the English-language literature. The research results are intended to serve as a basis for the verification of the initial assumptions made in the authors’ planned research work, while their compendium is presented in this review article.

The aim of this paper is to characterize the most popular binders used in the stabilization/solidification of heavy metal-contaminated soils and to identify the most effective and environmentally friendly solutions currently in use. The above was achieved by analyzing the results of UCS compressive strength tests and information on the leachability of contaminants from S/S products presented in the literature. It should be noted that, due to the large amount of data reported and the volume of this article, the analysis of contaminant leaching was kept to a minimum and the focus was on the mechanical properties of the treated soil. At the same time, it is pointed out that a more detailed consideration of the binding mechanism of individual metals in soil–cement mixtures and the presentation of leaching test results achieved with the analyzed binders, as an extremely important issue, should be the subject of a separate paper. In addition, this article summarizes the research methods that can be used to assess the effectiveness and sustainability of the processes for strengthening contaminated soils while reducing the mobility of contaminants in the subsoil.

## 2. Samples Preparation and Curing Conditions

**Figure 1:**
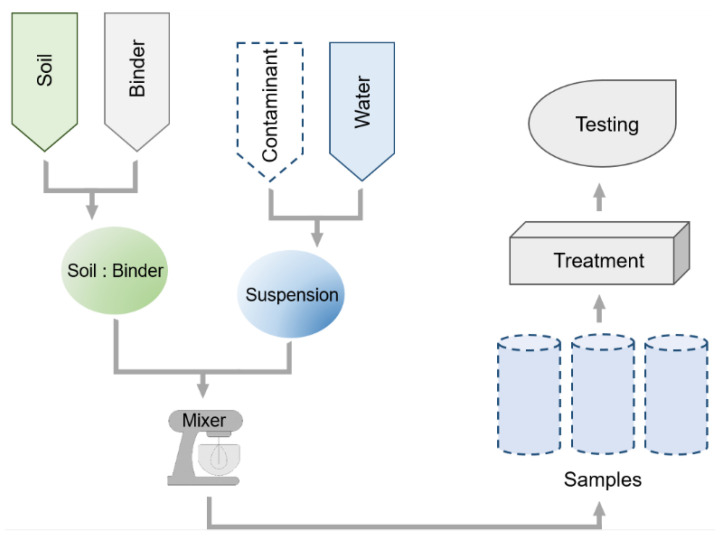
Scheme of the sample preparation procedure.

Essential criteria for conducting optimization studies of the S/S process are the repeatability and homogeneity of the samples. The standards describing the testing of cement–soil samples [27], as well as the literature data from published scientific results, can be used in this regard. The vast majority of the articles outlining sample preparation contain a scheme of the procedure, as shown in Figure 1.

The soil is initially dried to a solid mass, ground and sieved through a 2.0 mm sieve, and the selected additives dried to a solid mass are blended in appropriate weight proportions until a homogeneous mixture is obtained. A specified amount of water contains the assumed concentration of the contaminant, i.e., the selected heavy metal is added. Finally, mixtures with strictly controlled moisture content, composition and degree of pollution are obtained. The prescribed procedure is modified by various researchers. In Li and Poon’s study [28], contaminants in the form of lead nitrate were added directly to the dry mixture, before deionized water was fed. Goodarzi and Movahedrad introduced suspensions of the contaminant in distilled water into a dry soil sample, followed by blending the wet contaminated soil with binders [29]. However, a more correct approach appears to be to prepare the contaminated soil in advance, allowing for a thorough mixing of heavy metal ions and soil, as used in the study by Li et al. [30]. Soil with contaminants at a determined moisture content was stored under standard cure conditions for 30 days before being combined with binders at the assumed proportions. In the study by Wang et al. [31], contaminated soil taken from military sites was used; thus, an additional dosage of the contaminant was not required. In the case of the stabilization/solidification of wastes from the carbonation and hardening of steel, remediation was preceded by the initial inactivation of barium and cyanide by the addition of suitable chemicals, followed by blending with binders [32]. During the studies of Feng et al. [33], the binder was ground in order to increase its specific surface area, resulting in increased hydration. This step was carried out, despite the time-consuming nature of this method, up to a presumed degree of grinding and mixing of the ingredients, both dry parts and those with the addition of water, usually taking place mechanically at a preset time. The use of a planetary robot mixer is very common [31]. Alternatively, bench-top mixers are also used, where the mixer is inserted into a tall container filled with the mixture ingredients [34]. However, it is also common to mix the ingredients manually [32].

Filling the molds (cylindrical or cubic) with the mixture is usually performed in layers. For liquid consistency of mixtures, it is popular to use a vibrating table [35], which guarantees effective elimination of air bubbles. Alternatively, for mixtures with a plastic consistency, layers are applied with a constant compaction energy (manually [29], with the use of the Proctor apparatus [36] or other equipment with known compaction energy [37]). Few studies indicate the compaction of mixtures to the design’s volumetric density (e.g., 95% ρ_max_ [33] or to the maximum density of the soil skeleton [29]). The methodology mentioned above was found in all research papers analyzed. It should be mentioned that very few studies were carried out on a macro-scale, i.e., the S/S process is carried out in-situ and samples for testing are retrieved from the ground at an appropriate treatment time [38,39,40]. This obviously entails a lesser stability of conditions and homogeneity of samples, but allows actual environmental factors affecting the quality of the S/S process products obtained to be reflected.

The molded samples are subjected to a constant and very rarely modified method of curing. Immediately after preparation, they are wrapped in plastic film and stored for a suitable number of days at a stably maintained temperature of 20–25 °C (±2 °C) and a humidity level of 95% (±1%). The samples are unmolded either immediately or after 24 h (eventually 48 h), that is, after the initial setting of the mixture, depending on its consistency and sensitivity. Film protection and the maintenance of constant curing conditions are emphasized in all the studies analyzed.

The required period of curing corresponds to the assumed age of the samples at the time of testing. Usually, a series of samples is prepared for testing after 28 days. Often, 7-day-old samples are additionally tested, and sometimes (especially for the mixtures containing fly ash) 90-days old samples. Less frequently, however, 1-day or 3-day-old samples are tested. For the assessment of the extended setting time, part of the samples needs to be subjected to curing for several weeks.

By analyzing the data of the moisture content of the mixtures prepared for testing purposes, it was shown that the minimum amount of water applied to the dry weight of the soil, including the binder and any additives, is 7.5%, while the maximum is 60%. It is important to emphasize the significant influence of the initial moisture content of the mixture on the final results of the S/S tests [41]. However, very few sources in the literature present studies on the optimization of moisture content to obtain the maximum compressive strength of the S/S product with the different types of binders used. Some data can be found in the study by Kogbara et al. [36,42], which shows that contaminated sandy gravel subjected to the S/S process with Pulverized Fly Ash (PFA) and Ordinary Portland Cement (OPC) at a ratio of 4:1 achieves higher compressive strength as the moisture content of the mixture increases from OMC − 2% to OMC + 5%, where OMC denotes the optimum moisture content. In contrast, the opposite trend was shown in soil without contamination (sand stabilized with PFA and OPC in a ratio of 5:1) [43], where the maximum strength was reached on the dry side of OMC. Some information can also be found in the study by Boutouil and Levacher [44]. This very important aspect was analyzed by Kogbara in his paper [45], concluding, as a general rule, that the best mechanical and binding properties for contaminants were shown by the stabilized soils at a moisture content close to the optimum value, regardless of the binder system used. Due to the scope of this study, this important issue is considered to be an element suitable for a separate case study.

Wider variability is manifested in the amount of binder incorporated into the soil. In addition to the basic comparative (zero) samples, which consist of 100% dry matter soil and 100% dry matter binder (cement or as a mixture with another binder), soil/binder mixtures are being established. Soil accounts for 10% to 95% of the dry matter. In line with ecological and economic considerations, the aim is to minimize the amount of binder used in the S/S process, while maintaining the high efficiency of this method in binding contaminants and obtaining sufficient strength characteristics. Such a compromise is achieved through the use of various activating additives, water reducers or waste materials with unique properties, presented later in this article. It is also sought to replace as much Portland cement as possible with alternative pozzolanic materials, mainly waste materials (along with possible additives). A binder-to-soil ratio of 20–35% is considered to result in the achievement of immobilization of contaminants in the soil at a level acceptable to legislation [46]. In terms of varying the amount of Portland cement in relation to other binders, the amount of alternative materials in the studies analyzed varies from 10 to as much as 100%.

## 3. Characteristic of Binders, Amendments and Additives

**Figure 2:**
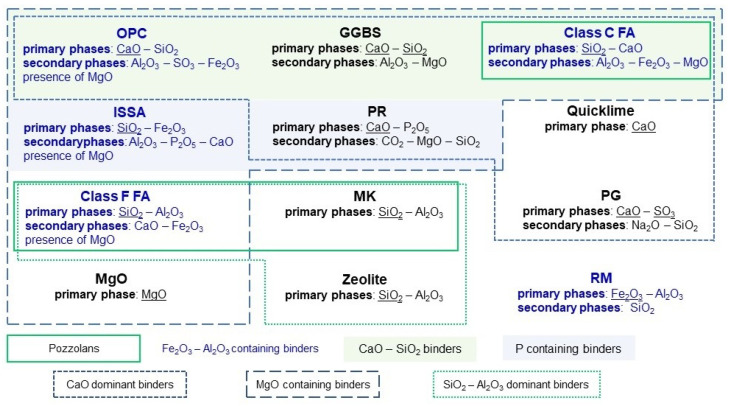
Classification of binders and additives for the stabilization/solidification of soil contaminated with heavy metals.

**Figure 3:**
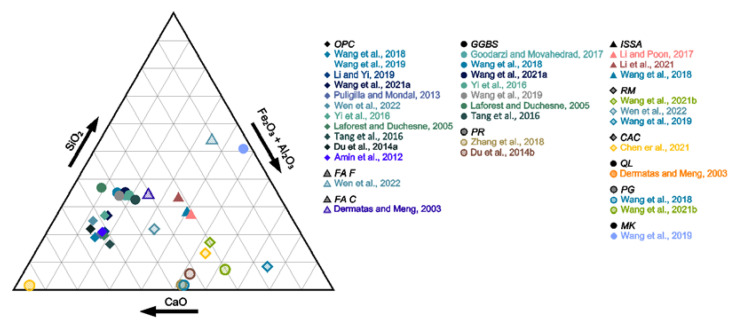
The ternary CaO-SiO_2_-Al_2_O_3_ + Fe_2_O_3_ diagram of selected materials analyzed in the paper based on Table 1 [12,28,29,31,37,50,57,58,59,60,61,62,63,64,65,66,67,68,69,70].

From a geotechnical point of view, the addition of the binder is expected to result in improved parameters of the subsoil for future engineering usage. However, the presence of contaminants (e.g., heavy metals) results in the need to select a binder that will additionally have an environmentally positive effect on mobility and bioavailability of contaminants [47,48]. In addition to the most commonly used binder, i.e., ordinary Portland cement, waste materials such as ground granulated blast furnace slag [34], incinerated sewage sludge ash [49], fly ash of different classes [33], phosphogypsum [38], and red mud [50] were proposed. This approach has a positive environmental impact due to the increased recovery of waste, which is in line with the trend of a closed-loop economy. Additionally, natural materials in their raw or modified state were utilized, e.g., bentonite [51], phosphate rock [52], lime [47], quicklime [38], metakaolin [53], magnesia [54] and hydrated lime [55]. Among the materials mentioned, fly ash and metakaolin can be classified as pozzolans, silicates or aluminosilicates, which, in the presence of water, take part in a reaction with lime giving the end product in the form of insoluble components [56]. The content ranges of individual phases and a graphical representation showing the grouping of materials by chemical composition are shown in Table 1 and Figure 2 and Figure 3, respectively.

The presence of individual phases affects not only the bonding process of the binder, but also the nature of the processes involved in the immobilization of contaminants. For example, the presence of phosphorus in ISSA can potentially affect the transformation of metals to insoluble forms of phosphates [67], and the presence of iron oxides (e.g., in red mud) can result in the transformation of metals to reducible forms [48]. Additionally, in order to ensure the proper course of the stabilization/solidification processes, the use of various types of activators was suggested (such as reactive MgO [29,55,57], cement [29] or hydrated lime [55] as an additive to GGBS), or mixtures of binders, additives and/or activators (such as fly ash and slag with the activator solution [58] or magnesium potassium phosphate cements mixed with FA or GGBS [73]). Zhang et al. [66] proposed a new binder produced on the basis of oxalic acid-activated phosphate rock and Du et al. [69] additionally enriched this material with monopotassium phosphate and reactive magnesia, which can be applied to Zn and Pb contaminated soils with elevated concentrations. The use of an activator (e.g., oxalic acid) allows controlling one of the most important determinants of the reaction, namely the relatively low pH that reduces the solubility of phosphate and metal compounds (e.g., Pb) [74]. Moreover, Li et al. [67] applied a similar activation method (using oxalic acid) of incinerated sewage sludge ash to immobilize high concentrations of Pb (5000 mg/kg). Considering the wide variety of approaches proposed in the literature, detailed characteristics of the most commonly selected materials for stabilization/solidification of contaminated soils, mainly in view of soil reinforcement, are presented in the following section of the paper. The materials potentially suitable for stabilization/solidification of contaminated soils, which have not found wider interest in practice, are not included in the paper. Examples of such materials include paper ash [75], high belite sulfoaluminate cement [59], municipal solid waste incineration fly ash [70], silica fumes [49,76,77], calcium carbide residue [78] and calcium sulfoaluminate cement [79]. However, this paper includes a short section dedicated to SPC binder, which, despite its lack of presence in the literature, is an environmentally friendly composite material that achieves very good strength and leachability results, and, thus, represents an interesting solution that can be analyzed in the future [72].

### 3.1. Ordinary Portland Cement (OPC)

The main binder used in soil stabilization/solidification is Portland cement OPC [45,80,81,82,83,84,85,86,87,88,89,90]. It is known to be highly effective in binding contaminants, as determined by numerous leachability tests and geochemical modelling [13]. It has been claimed [91,92,93] that heavy metal ions precipitate to form hydroxides and are captured by hydration products due to the alkaline environment. However, studies have provided neither direct evidence for its validity, nor sufficient reasons to refute it [94,95,96,97,98]. Instead, based on the results of the study, the probable adsorption of heavy metal ions on the surface of the C-S-H complex was indicated [99]. In addition, through XANES and Raman spectroscopy, it was found that the S/S process resulted in the incorporation and/or adsorption of lead onto silicate and calcium hydrates and onto ettringite [100]. Although Portland cement is still the primary binder for the stabilization/solidification of contaminated soils [101,102], the disadvantages associated with its use are well known. Some inorganic impurities, including heavy metals, interfere with the hydration of this binder, so that the engineering characteristics of S/S products are reduced [103,104,105,106,107]. To counteract this, an increased amount of OPC is used in relation to the soil, but this entails a significant consumption of energy to produce it and, thus, also creates a high carbon footprint [108,109,110,111]. In addition, the use of cement is fraught with uncertainty regarding the long-term effectiveness of soil binding due to its low chemical compatibility with the soil [46,112,113,114], its susceptibility to erosion by sulfate [56], acid rain [115], freeze-thaw cycles [116], and the predicted reduction in material characteristics over time [117]. These are the main reasons for seeking alternatives to cement among waste materials that have good pozzolanic properties. Due to the significant effect of the pH of mixtures undergoing the S/S process on the leachability of heavy metals [82], wastes that cause a slight reduction in the pH of the strongly alkaline environment created during cement hydration are particularly desirable.

### 3.2. Ground Granulated Blast-Furnace Slag (GGBS)

A widely used substitute for parts of Portland cement is ground granulated blast furnace slag GGBS, which is an industrial by-product of steel production [53,118]. An efficient reaction with calcium hydroxide during cement hydration forming an insoluble gel (C-S-H) is proven [119]. In addition, due to the higher specific surface area of its particles, GGBS leads to the formation of more hydration product nuclei [120]. This waste binder reduces the excessive increase in pH, thus affecting the solubility of heavy metals (most metals show the lowest solubility in solutions with pH~10), while having a positive effect on the final strength of the S/S product [31,121,122,123]. To mitigate the effects of heavy metals (e.g., lead) on cement hydration, phosphate- and sulfate-rich materials are often incorporated into mixtures along with GGBS for further stabilization of the pH value of the soil–cement mixture at the required level [32,124,125,126]. The materials of this type include the incineration sewage sludge ash ISSA or phosphogypsum. It is also known that GGBS activated with sodium compounds (NaOH, Na_2_SiO_3_, Na_2_CO_3_) with strongly alkaline properties causes an excellent increase in the compressive strength of soils [127,128], proving the beneficial effect of high pH during hydration on the final mechanical properties of the substrate, but without the presence of contaminant. The study also established that the supply of additives in the form of sulfates (e.g., salt—sodium sulfate) should be strictly controlled. Research results are available that show very good immobilization of barium and improved mechanical properties with Na_2_SO_4_, but also a decrease in compressive strength of the S/S product with excessive dosage of this compound [129]. GGBS is also combined with activators in the form of magnesium [130] or calcium oxides, which result in the formation of more hydrotalcite-like phases [131], which, in turn, have a beneficial effect on product strength. Magnesium and calcium oxides have many other advantages, described in a separate section of this paper. An important feature of GGBS is also its favorable grain size for the S/S process. As a fine-grained material, it fills the pores, thickening the structure and, thus, seals the subsoil, further immobilizing heavy metal pollutions [118,132]. It also decreases the porosity of cement pastes [63] and increases the density of the S/S product [133]. This type of binder is proving to be more effective than cement in Pb [46], as well as Cr immobilization, but only if the impurity is present in concentrations of no more than 2000 mg/L [62]. GBBS compounded with cement at a ratio of 1:4 also shows similar or better effects on geogenic As-containing soils than cement without any amendments [134]. Furthermore, GGBS is used in the production of geopolymer-based cement and recycled aggregate (GRAC) [135], where, with fly ash, it is a substitute for part of the cement. Nevertheless, GGBS is very frequently combined with cement, or, alternatively, lime. However, studies have indicated low compressive strengths achieved by S/S products using GGBS and lime [136].

### 3.3. Incinerated Sewage Sludge Ash (ISSA)

Studies of soil stabilization/solidification using ISSA and cement mixtures have indicated the high effectiveness of fly ash of this type in the adsorption, precipitation and physical immobilization of contaminants [28]. Due to the ability of the S/S product structure to buffer acids, there is an incorporation of metals into the soil structure in hydrated form, which manifests itself in a lower concentration of the harmful form of metals (lead, zinc, copper) [137]. When ISSA was used as an additive to a binder composed of OPC and GGBS, very good strength results and values competitive with other additives were obtained in leachability tests, but only at a certain molar ratio of additive to contaminant [31]. At molar ratios of 1:4 and 1:8, an excessive increase in the amount of ISSA was necessary and this resulted in an unacceptable decrease in the compressive strength of the S/S products. Stabilization/solidification studies using ISSA have also been carried out with the incorporation of an activator in the form of oxalic acid (OA), which has been successfully used in the activation of phosphate rock [67]. The aim of the addition of phosphoric acid is, in this case, for the activation of ISSA to release phosphates that react with lead to form lead phosphate hydroxides. This activator increases the effectiveness in stabilizing Pb, but its overuse can lead to the leaching of phosphate and zinc from the ISSA into the subsoil, so mixtures with these two components should be designed very responsibly.

### 3.4. Fly Ash (FA) and Pulverized Fly Ash (PFA)

Fly ash, as an industrial by-product, is readily used as a binder for the S/S process. For many years, it has been used as a replacement for parts of Portland cement in soil stabilization/solidification [39,42,88,117,129,138,139,140], especially in lead stabilization, due to its greater efficiency compared to the use of cement alone [141]. Both Class C fly ash [88] and Class F fly ash [142] are used for soil stabilization/solidification. According to ASTM C618 [143], the two classes differ primarily in the sum of SiO_2_, Al_2_O_3_ and Fe_2_O_3_ contents, and calcium content. There is a lack of studies in the literature optimizing the most efficient PFA:OPC ratio, most likely due to the disproportionate amount of research effort relative to the potential benefit [46]. A ratio in the range of 1:1 to 4:1, which is usually adopted, allows satisfactory results to be obtained from the studies, verifying the effectiveness of the S/S process [144]. It should be emphasized that most PFA cannot be used as a separate binder due to their too limited free CaO content for self-hardening purposes [145]; hence, the addition of OPC is necessary, usually in an amount not less than 20% of the ash quantity. Attempts have also been made to combine PFA with lime instead of cement [146], achieving very high unconfined compressive strengths of the S/S product. [68]. In addition, it is known that high-calcium fly ash (CFA) performs very well in silicate–aluminate–phosphate geopolymerization with magnesium phosphate cement (MPC) [73]. Information concerning MPC is included in a separate section of the study. The effect of the addition of 300 g (per kg of waste) of Na_2_SO_4_ to the mixtures in which the waste was stabilized with fly ash with cement (25:75 ratio) and slag cement was also studied. The introduction of salt resulted in a noticeable increase in 28-day unconfined compressive strength [32]. The referenced study also showed an increased the binding efficiency of cyanide and barium contained in the stabilized waste.

### 3.5. Magnesium Potassium Phosphate Cement (MPC)/(MKPC)

Magnesium potassium phosphate cement is a clinker-free acid-base cement [147], which is characterized by its high strength, resulting from the reaction of MgO (dead-burnt magnesia DBM) with potassium dihydrogen phosphate, during which amorphous or crystalline k-struvite and crystallized bobierrite are formed [148,149]. In addition, chemical stabilization of lead occurs due to the reaction with residual phosphates, during which lead phosphate and pyromorphite precipitate [103]. MPC exhibits greater chemical stability than OPC, which manifests itself, among other aspects, by minimizing the adverse effects of lead on the unconfined compressive strength [150]. MPC is further used to stabilize low-activity nuclear waste (containing plutonium, neptunium, caesium, strontium, actinium, technetium and selenium) as well as cadmium, chromium, copper, nickel, lead and zinc from electro-waste [151,152,153]. On the basis of an analysis of the test results available in the literature, it was also found that zinc ions reduce the degree of MPC cement mineralization, although they do not affect the hydration phases of the cement [154].

### 3.6. Red Gypsum (RG)

Another binder used as an amendment of OPC in the S/S process is red gypsum, a by-product of the production of titanium dioxide (a white pigment widely used in the food and cosmetics industries). In combination with OPC, PFA and GGBS, and a small addition of lime, mixtures with satisfactory compressive strengths were obtained. The values turned out to be lower than those of the soil combined with cement, but higher than the assumed minimum values (0.350 MPa) [155]. In light of the test results, the gypsum-GGBS binder was considered as an environmentally friendly alternative to OPC, guaranteeing sufficient mechanical properties of the S/S product.

### 3.7. Phosphate Rock PR and Phosphoric Acid (PA)

Phosphate rock and phosphoric acid have the potential to convert the lead present in soil into stable forms, such as pyromorphite. For this reason, they are widely studied and even recommended for use in the stabilization/solidification of soils contaminated with this heavy metal [19,47,70,156,157]. The effectiveness of phosphorite as an additive in binding zinc is also confirmed [158,159]. When using phosphates in the S/S process, the acidic environment required for their effectiveness must be taken into consideration [69]. In addition, it is pointed out that the grade of phosphate in the ore is not consistent [66] which, for obvious reasons, affects the effectiveness of this material in the S/S process. For these reasons, researchers designing soil mixtures with stabilizing/solidifying additives often opt for the implementation of a phosphate rock activator in the form of phosphoric acid. Other additives are also used in conjunction with this raw material, including monopotassium phosphate and reactive magnesia, forming a binder indicated by the KMP symbol. Studies have confirmed the rather high early strength of KMP-stabilized products due to the hydration rate of reactive magnesia [69], as well as the possibility of increasing strength and decreasing leachability when KMP is used together with an accelerated carbonation process [160]. The aforementioned process, also investigated for reactive magnesia (in a separate part of the paper), is introduced as a factor corresponding to an accelerated version of natural weathering [161].

### 3.8. Phosphogypsum (PG) and Potassium Dihydrogen Phosphate (KDP)

Phosphogypsum is a by-product of phosphoric acid production, containing calcium sulfate and phosphate residues. As a phosphate-rich material, it exhibits similar properties to potassium dihydrogen phosphate KDP and ISSA, i.e., it reduces the excessive increase of pH of the soil–cement mixture. Thus, during hydration of the cement or another binder, conditions are unfavorable for the dissolution of metals [28,124,162]. Both PG and KDP are used in the S/S process mainly as additives to reduce the reactivity of contaminants. PG has been combined, among others, with Basic Oxygen Furnace Slag (BOFS) and Calcium Carbide Residue (CCR), the total composition of which is rich in dicalcium and tricalcium silicate, CaO, CaSO_4_·2H_2_O, and CaCl_2_, as well as phosphates, fluorides, and sulfates, to ensure that binder hydration can develop in contaminated soil [33].

### 3.9. Red Mud (RM)

Red mud is an alkaline by-product of the Bayer process, taking place during the production of aluminium (bauxite refining) [163]. It forms a toxic and corrosive sludge with a high pH and very high iron oxide content. It has been applied to the S/S process to stabilize heavy metals (lead, zinc and cadmium) as an additive to phosphogypsum and Portland cement [50]. A binder of this composition shows sufficient strength and binding characteristics for metals in the stabilization/solidification process at landfill sites, while also contributing to the recycling of red sludge. It has also been used successfully to reduce the bioavailability of Cd, Pb and Zn [163].

### 3.10. Calcium Aluminate Cement (CAC)

Calcium aluminate cement is a relatively rarely studied binder in the stabilization/solidification of soils contaminated with heavy metals. It is more often included in the remediation of soil contaminated by organic pollutants with possible accompanying inorganic pollutants. On the basis of a study by Contessi et al., it was found that CAC exhibits a completely different mechanism in the immobilization of lead than is the case with the use of OPC [99]. Due to its high sulfate content, the reactivity is strongly shifted towards the formation of ettringite, in which case, Pb^2+^ ions are incorporated into its structure, in place of some calcium ions. The results obtained by Bougharraf et al. [164] led to the conclusion that CAC was more effective in binding organic contaminants compared to inorganic contaminants. At the same time, the binding of heavy metals was determined to be satisfactory, especially with an achieved compressive strength higher than 1 MPa. In other studies, the retention of metals (lead, copper, zinc) using CAC was determined at a level of 99.9% [165], and chromium in the form of Cr^6+^ at a level exceeding 90% [166], which was a better result compared to using OPC. However, in addition to the leachability results, the strength tests for the S/S products using calcium aluminate cement are above average. Depending on the amount of binder addition, the strength is higher than when using sulfate-resistant Portland cement from about 0.5 to even 4.0 MPa [167]. Despite the excellent strength results and the leachability of contamination, the use of CAC is very limited, due to the high cost of this raw material.

### 3.11. Bentonite

Bentonite is an additive to various types of binders in S/S processes. It has been found to affect the decrease in strength of the S/S product, but is excellent at improving the degree of immobilization of contaminants, both organic and inorganic [164].

### 3.12. Lime (CaO), Quicklime (QL), Lime Production Waste (LPW)

Lime is a material mainly used for soil stabilization. When added to soils with a high content of clay particles in the presence of water, it undergoes cation exchange and further reactions. As a result of the modification of the electrical charge density, the forces of intermolecular attraction increase, which directly results in a strengthening of the soil structure [3] and a favorable change in the Atterberg limits [168]. The long-term effectiveness of lime stabilization of loess has also been proven [169], which is extremely important for the design of permanent structures on this type of subsoil. Furthermore, hydrated lime is an activator for GGBS-type binders [170]. CaO proves to be very effective as an additive to GGBS at a ratio of 1:9, as demonstrated in the studies of contaminated soils subjected to S/S, performed after 18 months of treatment [170]. It has also been successfully used as an activator at a ratio of 15% to 85% GGBS [54] in the S/S process of soils contaminated with lead and zinc. It is also used as an additive to red gypsum (RG), GGBS and PFA to increase the alkaline pH of the mixture, resulting in better hydration and, consequently, higher strength [155]. In a study by Moon and Dermatas, it was also proven to effectively bind lead to acceptable levels using Quicklime and FA [171]. Lime production waste (LPW), obtained if the raw calcite or carbonate materials undergo burning at temperatures lower than 960 °C, is a by-product containing lime, Al_2_O_3_, MgO, Fe_2_O_3_ and other components, which is also of interests in soil stabilization, especially in mixtures with blast furnace slag and red mud [12].

### 3.13. Reactive Magnesia (MgO)

Magnesium oxide is usually not used as a separate binder, but as an additive to improve the properties of binder materials other than cement [172,173]. Its main advantage is to increase the effectiveness of binders in immobilizing contaminants, both inorganic and organic [16]. MgO facilitates the transport of the strongly alkaline binder to the work site, and counteracts excessive settlement of the substrate during the S/S process, thus increasing the cost-effectiveness of its application [29,55,131]. In addition, it increases the neutralizing properties for an acidic environment, and this is an extremely valuable feature when the S/S process substrate is exposed to acid rain [34]. In addition to the widespread use of MgO as a binder additive, experimental processes to stabilize/solidify contaminated soil using only reactive magnesia have also been carried out; however, the unconfined compressive strengths of the product S/S obtained were 80–90% lower than those obtained using OPC [16,70]. For this reason, an additional factor, i.e., carbonation, was incorporated into the blends under development [174,175]. Carbonation was carried out after the mixture was placed in the molds. To test the effectiveness of this solution for lead- and zinc-contaminated soils, comparative UCS and leachability tests, were conducted for the mixtures stabilized with MgO and CO_2_ injection, and for the mixtures stabilized with OPC. In each case, only 5% binder dry weight and 95% soil dry weight were used. The samples containing OPC were tested after 28 days of treatment, while samples treated with MgO and saturated with carbon dioxide were tested after 72 h (lead-contaminated) and 120 h (zinc-contaminated). CO_2_ was found to cause relatively high variability in the unconfined compressive strengths achieved by S/S [175,176], but at the same time, at Pb and Zn concentrations > 4000 mg/kg dry weight of soil, the strengths were significantly higher than those achieved with OPC.

### 3.14. Geopolymers and Geopolymeric Binder Made of NaOH-Activated Metakaolin (MK)

Great hopes were entertained in geopolymers for an environmentally friendly cement substitute, also for use in the S/S process. In addition to its high unconfined compressive strength, this innovative binder provides high resistance to acidic and high temperature environments, low permeability and extended durability [177,178,179,180,181]. However, despite intensive research and continuous development, this material has failed to revolutionize the binder segment. While the strength and leachability results of S/S products are competitive with those using traditional binders [182,183], the costly activators that enable the synthesis of the geopolymers (sodium or potassium hydroxides, liquid water glass) block their widespread use. The situation is partly alleviated by the fact that geopolymers based on slag [184] and fly ash [58,185] are currently being produced and studied in addition to metakaolin-based geopolymers [99,186]. These raw materials, as waste materials, increase the ecological value of the solution, which, in addition to very good characteristics, opens the possibility of its dissemination. However, the implementation of geopolymers has been quite limited so far.

### 3.15. Natural and Synthetic Zeolite

Both natural and synthesized zeolites from fly ash are known for their very good sorption properties, thus gaining reputation as additives that enhance the immobilization of contaminants in the S/S process. These properties are used towards the binding of petroleum substances [187] and inorganic substances in the form of heavy metals [188]. Due to the specific structure of zeolites rich in voluminous free spaces, it is possible to encapsulate large cations, such as sodium, potassium, barium, and calcium, and even molecules and catalytic groups of water, ammonia, carbonate and nitrate ions [189]. Furthermore, during the study, it was found that, due to the large reaction surface area of zeolite, resulting from its significant porosity, the S/S process using this amendment combined with OPC achieves higher unconfined compressive strength values and greater durability [190]. It was also proven that the high cation exchange capacity and high content of clinoptilolite, as well as the low concentration of potential toxic elements, cause a high potential for treating industrially contaminated soil with use of thermally treated natural zeolite [71].

### 3.16. SPC Binder

The reaction of single superphosphate (SSP) and calcium oxide (CaO), used simultaneously in the S/S process, leads to the formation of hydroxyapatite (HA) in the soil structure. The combination of SSP and CaO, defined as the SPC binder, thus provides benefits in the form of a densification of the structure and an increase in the unconfined compressive strength of the treated soil. In parallel, it offers an alternative to a synthetic hydroxyapatite, which, despite its many advantages, is a material that is too expensive for widespread implementation in the S/S process. Meanwhile, the environmentally friendly SPC binder, at a relatively low cost, causes the formation of calcium-phosphate crystals (including HA), which form strong cementation bonds between soil particles, and the various reaction products fill the pores (especially inter-aggregate pores) [72]. In addition, studies were performed on in situ stabilized/solidified soil with use of the SPC binder, which confirmed the good results shown during laboratory testing [40].

### 3.17. EnvirOceMTM

For solutions to local contamination problems, targeted mixtures are being developed for a given soil type and type of contamination, as in the case of the remediation of the former Astra military explosives Fireworks site in SE England [191]. A subsoil contaminated with high concentrations of zinc, lead and copper was subjected to an S/S process using superfine sulfate-resisting Portland cement, functioning under the name EnvirOceMTM. Satisfactory leachability results were obtained, both after 28 days and after 4 years of the S/S process. Unfortunately, the compressive strength of the S/S product was not verified.

## 4. Effectiveness of Mixtures and Optimization Testing Methods

The effectiveness of the S/S process is defined by the geomechanical parameters and the leachability of its products. The applicability of the solution is also strongly influenced by its durability. These properties are verified by analyzing the results of specific tests, the main types of which are summarized in this paper. Preliminary tests, which were used by the authors of the cited publications to describe the components of the designed mixtures, such as mineralogical composition, specific density, grain size, etc., were not systematized. At this point, it should be emphasized that research results indicate that no single binder can perfectly remediate all types of heavy metal-contaminated soils, and binder selection is a site-specific problem [192]. With the above in mind, a specific soil contaminated with a particular heavy metal should be tested to determine the best binder combination for stabilization/solidification purposes using data and experience described in the literature for similar cases. In contrast, valuable studies have been reported in the literature, with design charts directly indicating the proportions of water and binders in regard to the performance criterion of the S/S process of similar soils contaminated with the same heavy metals [42].

### 4.1. Unconfined Compression Strength

The geomechanical properties of a S/S product are mainly represented by unconfined compressive strength, and the test used and performed on mixture samples is the uniaxial compression strength test (UCS), which can be conducted according to ASTM [193]. It is conducted as a standard for both soil stabilization [194] and the S/S process. In almost all sources analyzed, the strength is tested after 28 days of treatment, often also after 7 and/or 90 days; the testing of 1-, 3- or 160-day samples is rare. The age of the test specimens is mainly determined by the mixture formulations, especially with regard to the fly ash content, which generally reaches full strength later. The results of the UCS tests are summarized for comparison purposes in Table 2. 

### 4.2. Leaching Behavior

**Figure 4:**
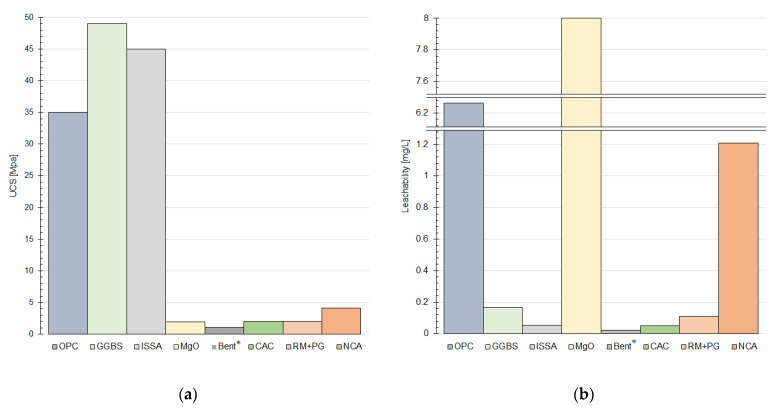
A few selected test results based on analyzed data: (**a**) unconfined compressive strength; (**b**) leachability. * Bent: Bentonite.

The Toxicity Characteristic Leaching Procedure (TCLP) is the most widely used test by which researchers determine the binding efficiency of contaminants in S/S products. Performed according to the procedures described in US EPA standards [196], it allows the comparison of effectiveness between different types of mixtures, including studies performed by different authors. Nonetheless, the TCLP test is not considered adequate by all authorities in the field of S/S testing. TCLP is mainly dedicated to the analyses of the leachability of contaminants from landfill waste, and does not reflect the conditions of the natural subsoil [167,197]. For this reason, some researchers use the Simulated Precipitation Leaching Procedure Test (SPLP) [198] instead, which allows determination of the leachability of contaminants from the ground into groundwater [28]. However, they are in a distinct minority, so the results from the more popular TCLP-type test were adopted for comparison. The data given in Table 2 were compiled from an analysis of the leachability test results presented in the papers studied. The leachability potential of contaminants from an S/S product can be estimated in two different scenarios: in the diffusion process, the most likely way corresponding to reality (semi-dynamic tank leaching [199,200]); or after grinding the product immediately before testing, which obviously maximizes the leachability of contaminants and corresponds to the worst possible scenario (Batch Leaching test [201]). In addition, contaminant leachability tests at varying pH, as well as the Acid and Base Neutralization Capacity (ANC/BNC), are used, depending on the scope of testing and the planned use of S/S products. The hydraulic conductivity test, which captures a picture of the water filtration capacity of the treated subsoil, should also be considered. In the case of many binders, the binding of contaminants also takes place by physical immobilization, so that an open filtration channel would allow the leaching of contaminants into the deeper layers of the subsoil and, consequently, into the groundwater. For this reason, hydraulic conductivity is closely related to leachability and is often tested as an indicator of process efficiency [46]. Another test that gives a picture of the leaching of contaminants with solutions of increasing aggressiveness is the BCR SEP (The Community Bureau of Reference Sequential Extraction Procedure). It allows the quantification of exchangeable fractions, reducible fractions, oxidizing fractions, and heavy metal residues in the soil. The test procedure described by Davidson et al. [202] has also been adopted in tests by other researchers. A quantitative description of the chemical stability of heavy metals in soil is possible by adopting a selected variable with an appropriate formula, such as the relative binding intensity (IR) index. S/S products are also studied in terms of pH, which is one of the factors shaping the leachability of heavy metals [203]. There are known results indicating that the lowest leachability of lead and zinc occurs when the pH of the product approximates 9.5 [204,205]. Individual countries regulate the maximum contaminant level (MCL), which corresponds to the concentration allowed in drinking water, by means of internal regulations. Due to the differences between the laws and the different ranges for individual metals, this study indicates the maximum allowed value for lead after Toxicity limitation (US EPA) standard (5 mg/L) and for zinc after Toxicity limitation (GB5085.3-2007) (100 mg/L). As mentioned in Section 4.1, a comparison of the results of individual reported studies of both UCS and leachability is not representative, and, furthermore, due to the large number of variables, impossible to present in a single chart. For this reason, Figure 4 includes data from only a few selected literature entries using different binders to show the degree of variation in the results obtained.

### 4.3. Microstruture Investigation

Determining the changes that have occurred in the microstructure of the soil as a result of the binder introduced requires specialized procedures and very sophisticated equipment. It is also necessary to have detailed knowledge in order to be able to analyze the results in terms of the resulting chemical products, such as phase hydration products or precipitated heavy metal compounds. A common microstructure test to determine mineralogical composition is X-ray diffraction [7] and thermogravimetric analyses [126]. Hydration heat evolution testing [103] is also implemented. In addition, scanning electron microscopy (SEM) and transmission electron microscopy (TEM) are used as confirmatory and refinement imaging tests for XRD-type studies. The TEM technique is particularly useful for detecting the presence of the cemented phase and the pozzolanic phase, i.e., by determining the C-S-A-H (calcium silicate aluminate hydrate) bonds present [3]. It is also important to determine the microporosity of the S/S-treated subsoil [33], which is made feasible by the mercury intrusion porosimetry (MIP) test [69]. Analysis of the MIP test results allows prediction of the mechanical behavior of the solidified soil and the extent to which its pores are filled by stabilization products.

### 4.4. Electrical Resistivity

Electrical resistivity EC is a criterion that has been used over the years both in assessing the hydration process of cement pastes [206], and the mechanical and deformation properties of soils [207,208]. Its immense advantages for use in geotechnics and geoengineering are its relatively low cost and time-efficient testing and, above all, its lack of interference with the natural soil structure [209]. The work of Liu et al. showed that the electrical resistivity of soil–cement mixtures can be correlated with the strength (*q_u_*) of UCS with a good fit [210] in an exponential function [211]. On the other hand, the study of Chen et al. gave rise to the conclusion that as the concentration of lead contamination in the soil increases, a lower magnitude of electrical resistivity is obtained, while it increases with the time of the treatment process and the binder content of the mixture [101].

### 4.5. Durability

Durability is understood as the degree to which the mechanical and bonding properties are retained after an adverse ageing factor, e.g., drying–wetting cycles, freeze–thaw cycles, sulfate attack or acid attack, as well as exposure of the S/S product to an environment with average erosion properties over a period of several years [212]. Due to the potential impact of acid rain, freeze–thaw, drying–wetting and other environmental factors, it is necessary to know the long-term effectiveness of the solution used in the S/S process. The types of tests listed below reflect adverse natural phenomena that can reduce the originally achieved S/S product characteristics.

#### 4.5.1. Drying–Wetting Cycle

One of the ageing factors to which the products of the S/S process are subjected is the drying–wetting cycle. During this process, soil–cement specimens are alternately heated in an oven for 48 h and at a preset temperature (30–40 °C), followed by soaking in distilled water at a stably maintained temperature over 24h [35,131]. During testing, the weight loss after each 72-h cycle and the compressive strength are usually checked. The strength is compared with that of a control test, in which the specimens are cured under stable conditions, as described in point 2. of the paper. Data can also be found on the compressive strength and leachability of specimens subjected to this ageing factor. Depending on the type and purpose of the test, it is possible to modify any of the test conditions, e.g., soaking in water with a reduced pH, corresponding to acid rain, or being subjected to drying at a higher temperature than actually prevailing in summer (60 °C for 24 h) [50]. Basic test guidelines can be found in the ASTM Standard [213].

#### 4.5.2. Sulfate and Acid Attack

The ageing effect of cements, and consequently of cement–soil mixtures, is also influenced by the chemical composition of the environment in which it occurs. Both sulfate and the acidic pH of liquids negatively affect the strength of cement, mainly due to the degradation of C-S-H bonds [214]. The cements based on waste materials activated with MgO show a slightly lower sensitivity [173]. In order to determine the magnitude of this effect, samples are placed in suitable solutions, e.g., 5% Na_2_SO_4_ or, alternatively, acid, for a period of several tens of weeks [53,179]. The impact of acid rain can also be mapped in TCLP studies using an extraction liquid with pH = 2.0 to 7.0 [34,115]. There are no general methodological guidelines for testing the effects of sulfates and acids on the strength of concrete or S/S products, and both the scarcity of research in this direction and the need for its development is emphasized by all authors addressing this challenge [215].

#### 4.5.3. Freeze—Thaw Impact

The effect of varying temperatures is important in the near-surface layers of the subsoil, where its fluctuations have a real impact on product S/S. This is reflected in freeze-thaw cycles, during which a cement–soil sample is placed in an apparatus dedicated to this test [116]. Alternatively, the soil sample is transferred from the freeze–thaw cabinet to a water container at a stably maintained positive temperature [50]. Regardless of the equipment used, the sample is subjected to alternating temperatures of −20 °C and +25 °C, with a change in ambient temperature every 24 h [116]. Basic guidelines for the test can be found in the ASTM Standard [216]. In a long-term study by Al-Tabbaa et al., 5-year old soil–cement samples with different binder types were subjected to 12 freeze–thaw cycles, during which the freezing temperature was set at −10 °C [39]. Similar to the drying–wetting cycles, the weight loss after each freeze–thaw cycle, as well as leachability and compressive strength, are checked in this type of study.

## 5. Conclusions

A subsoil that requires remediation must meet certain standards, due to minimum strength parameters and a maximum allowed leachability of contaminants. The analysis of research results presented in the literature enabled the determination of effective binder and additive mixtures in the stabilization/solidification of soils contaminated with heavy metals. Based on the analysis carried out, the following conclusions were drawn:

1. The variety of proposed binders, additives and their mixtures and methods of activating the materials is very extensive in the literature, providing engineers with a wide range of options depending on the geochemical conditions of the treated site.
2. Despite its many disadvantages, the most popular binder in the S/S process is Ordinary Portland Cement.
3. Implementation of waste materials such as GGBS, FA, ISSA as amendments for part of the OPC for the stabilization/solidification process is becoming common practice, with many environmental and economic advantages.
4. Replacing part of the cement with PFA or ISSA fly ash results in a significant decrease in the strength of the S/S product, but does not increase the leachability of the contaminants.
5. The implementation of GGBS in place of part of the OPC results in an increase in strength, but significantly increases the leaching of contaminants when used in too large a quantity. The addition of an activator (e.g., MgO) significantly improves the ability of GGBS solidification.
6. Considering the frequency of undertaking S/S process studies using red gypsum, red mud, calcium aluminate cement, bentonite, zeolites and superfine sulfate-resisting Portland cement, these materials should be considered niche products, effective for use only under specific conditions.
7. In optimizing the mixture of binders and additives for the S/S process of heavy metal-contaminated soils, one of the main factors considered should remain the ecological aspect.
8. The key studies assessing the effectiveness of S/S processes of contaminated soils are UCS and leachability studies. However, the scope of the latter varies widely and often does not take into account the actual conditions in the soil medium.
9. The often-overlooked ageing tests, which take into account the effects of external factors on the mechanical and chemical stability of the resulting bonds when assessing durability, should be important in the evaluation of the S/S method.

## Figures and Tables

**Table 1:** Chemical composition of binders and additives tested (wt%).

Binder/Additive	SiO_2_	Al_2_O_3_	SO_3_	CaO	TiO_2_	Fe_2_O_3_	MgO	K_2_O		P_2_O_5_	Na_2_O	CO_2_	MnO	F	LOI	Ref.
OPC	18.99–27.4	4.41–11.5	2.25–4.52	46.6–65.72	0.17–0.51	2.31–4.03	1.02–3.71	0.24–1.31	–	0.08–0.23	0.17–0.48	–	0.05–0.06	–	0–6.19	[31,37,57,58,59,60,61,62,63,64,65]
GGBS	32.7–36.77	7.77–29.43	1.46–2.37	31.49–40	0.36–1.63	0.31–5.54	5.5–13.91	0.43–0.85	–	−0.01–0.04	0–0.36	–	0.28–1.02	–	−1.49–2.67	[29,31,57,60,61,62,63,66]
ISSA	27.24–31.7	13.72–17.2	2.07–3.45	6.34–10.96	0.7–5.04	17.8–27.35	2.9–3.52	2.0–2.77	–	9.23–12.28	4.4–6.52	–	–	–	nt–0.99	[28,31,67]
Class F FA	53.97	31.15	0.727	4.01	–	4.16	1.01	2.04	–		0.89	–	–	–	–	[59]
Class C FA	34.2	19.3	2.2	25.8	–	5.64	5.07	0.52	–		2.4	–	–	–	0.11	[68]
PR	1.15–6.14	0.27–1.23	–	45.93–48.4	–	nt–0.16	nt–6.96	–	–	21.9 *–25.10	–	nt–13	–	2.23–2.41	nt–13.12	[66,69]
PG	1.26–8.8	nt–0.72	39.64–55.3	37.5–47.05	–	–	nt–0.32	nt–0.32	–	nt–0.03	nt–10.03	–	–	–	–	[31,50]
RM	9.11–21.43	4.57–26.1	nt-0.67	nt–45.15	nt–3.98	9.98–59.37	nt–0.33	nt–1.56	–	nt–0.37	nt–11.51	–	0.2–6	–	nt–13.41	[12,50,59,61]
CAC	7.38	52.9	0.31	34.1	2.23	1.83	0.37	0.45	–	0.19	–	–	–	–	–	[70]
QL	1.2	–	0.012	95.4	–	–	0.85		–	–	–	–	–	–	0.55	[68]
MgO	0.9–1.1	0.12–0.41	0.05–0.28	0.5–1.39	–	0.03–0.7	95.8–89.5	0.01–3.57	–	–	–	–	nt–0.02	–	0–2.76	[29,37,54,55,57]
MK	49.55–50.30	35.22–47	0.05–0.59	0.18–0.2	–	0.52–1.05	0–0.36	0.19–0.28	–	nt–0.28	0–0.01	–	–	–	nt–4.23	[61,65]
Zeolite	69.96	13.61	–	3.61	0.02	1.38	0.51	1.79	–		1.61	–	0.03	–	7.47	[71]
CaO	–	–	–	98.9	–	–	0.2	–	–	nt	–	–	–	–	–	[72]

* available 2.53; nt: not tested; LOI: loss on ignitation; OPC: ordinary Portland cement; GGBS: ground granulated blast furnace slag; ISSA: incinerated sewage sludge ash; FA: fly ash; PR: phosphate rock; PG: phosphogypsum; RM: red mud; CAC: calcium aluminate cement; QL: quicklime; MK: metakaolin.

**Table 2:** Abbreviated research results.

No	Symbol *	S/W	Binder/Additive			WC	Heavy Metal		UCS	Leaching	Ref.
		[%]	[%]			[%]	Type	[mg/kg]	[MPa]	mg/L	
1			PC	GGBS	ISSA	20	Pb	1941		TCLP	[31]
	S/PC	50	50	–	–				35	6.26	
	S/BC	50	25	25	–				49	0.168	
	Pb/ISSA = 1:2	50	18.10	18.10	13.8				45	0.055	
2			SC	OPC	ISSA	24–50%	Ba	168,000		Leachate pH	[32]
	C1	80	20	–	–				7.9	2.2	
	H3	50	–	37.5	12.5				8.6	4.3	
3			CAC/OPC/GGBS	TWEEN 80	BENTONIT	22	Pb	96.7		AFNOR NF X31-211	[164]
	M1	73	5/–/–	0.025	–				0.65	0.101	
	M2	68	10/–/–	0.05	–				2.0	0.05	
	M3	68	5/–/–	0.05	5				0.45	0.197	
	M4	63	10/–/–	0.075	5				0.95	0.143	
	M5	63	5/–/–	0.075	10				0.15	0.290	
	M6	58	10/–/–	0.1	10				1.1	0.022	
	M7	73	–/3/2	0.025	–				0.4	0.290	
	M8	68	–/6/4	0.05	–				1	0.068	
	M9	68	–/3/2	0.05	5				0.22	0.300	
	M10	63	–/6/4	0.075	5				0.95	0.105	
	M11	63	–/3/2	0.075	10				0.25	0.190	
	M12	58	–/6/4	0.1	10				1	0.068	
4			PC	MgO	CO_2_	7.5	Pb			Leached Pb [mg/kg]	[37]
	Pb + PC	95	5	–	–			4000	2.0	100	
	Pb + PC	95	5	–	–			16,000	0.9	1100	
	Pb + MgO + CO_2_	95	–	5	used			4000	1.9	8	
	Pb + MgO + CO_2_	95	–	5	used			16,000	1.95	9.8	
5			Ca(OH)_2_	MgO	GGBS	40	Zn/Pb			TCLP	[54]
	CGZn0.25	75	3.75	–	21.25		0.25		4.1	0.264	
	CGZn0.5	75	3.75	–	21.25		0.5		1.9	0.220	
	CGZn1	75	3.75	–	21.25		1		0.45	0.178	
	CGPb0.25	75	3.75	–	21.25		0.25		5.9	ND	
	CGPb0.5	75	3.75	–	21.25		0.5		7.9	0.072	
	CGPb1	75	3.75	–	21.25		1		7.9	0.18	
	MGZn0.25	75	–	3.75	21.25		0.25		4.9	0.091	
	MGZn0.5	75	–	3.75	21.25		0.5		5.0	0.082	
	MGZn1	75	–	3.75	21.25		1		3.2	0.076	
	MGPb0.25	75	–	3.75	21.25		0.25		5.0	0.066	
	MGPb0.5	75	–	3.75	21.25		0.5		5.9	0.062	
	MGPb1	75	–	3.75	21.25		1		7.1	0.166	
6			OPC	ISSA		20	Pb	5000		SBET [mg/kg]	[28]
	H0	90	10	–					10	36	
	H0.2	90	8	2					4.6	35	
	H0.5	90	5	5					2.6	31.5	
7	8	soil	OPC	GGBS		20	As	170.4		TCLP	[41]
	O5	96	4	–					3.2	0.011	
	O4G1	96	3	1					3.7	0.015	
	O2.5G2.5	96	2	2					6.0	0.021	
	O10	92	8	–					7.5	0.06	
	O8G2	92	6	2					7.8	0.065	
	O5G5	92	4	4					9.5	0.012	
8			RM	PG	OPC	nt	Zn/Pb/Cd	5000		TCLP	[50]
	RPPC7.5	92.5	4.3	1.1	2.1				0.726	4.2/0.8/8	
	RPPC10	90	5.7	1.4	2.9				1.1	0.8/0.3/0.9	
	RPPC15	85	8.6	2.2	4.2				2.013	0.3/0.11/0.7	
	PC10	90	–	–	10				3.3	0.4/0.9/0.8	
9			CCR	PG		16.5–17.6	Ni/Zn	6352/5352		China HJ/T 299	[33]
	10% bin/dos	90	6	3	1				0.46	0.01/0.12	
	<10%bin-dos	>90	<6	<3	<1				<0.3	>0.06/>0.7	
10			KSil	FA	KOH		Zn	15,900		TCLP	[183]
	KSil0.46KOH	21	16.8	58.8	3.4	18.8			0.75	8.67	
			OPC	FA	lime	22.2			2.0	<0.001	
	OPC lime	35.8	7.1	50	7.1						
11			NCA			13	Pb	10,000	*** 7d	*** 7d	[30]
	NCA ** 10%	90	10	–	–				4.1	1.21	
	NCA ** 20%	80	20	–	–				6.32	0.318	
	NCA ** 30%	70	30	–	–				10.12	0.075	
	NCA ** 40%	60	40	–	–				11.16	0.027	
12			SPC	–	–		Pb	9710		TCLP	[72]
		92	8	–	–	22			0.352	1.8	
		90	10	–	–	22			0.432	0.9	
13			OPC	GGBS	–	–	As	1985		TCLP	[134]
	O4G1	95	4	1	–	–			1.1	4	
	O2.5G2.5	95	2.5	2.5	–	–			1.05	5.3	
14			OPC	CaO	MgO	7.5	Pb	16,000		SBLT	[192]
	Pb + OPC	95	5	–	–				1.0	1000	
	Pb + CaO	95	–	5	–				0.18	8000	
	Pb + MgO	95	–	–	5				0.05	2	

S/W: Soil or Waste; WC: water content; TCLP: Toxicity Characteristic Leaching Procedure; SBLT: single batch leaching test; * Symbol of the sample after ref.; ** NCA: New curing agent, the composition of the binder is not specified; *** 7d: test conducted on the seventh day of treatment; GGBS: ground granulated blast furnace slag; ISSA: incinerated sewage sludge ash; PG: phosphorus gypsum; KDP: potassium dihydrogen phosphate; MKPC: magnesium potassium phosphate cement; MPP: mono-potassium phosphate; DBM: dead burnt magnesia; MPP + DBM = MPC; KMP: oxalic acid-activated phosphate rock + KH_2_PO_4_ + MgO [1:1:2]; SC: slag cement; CH: Ca(OCl)_2_; SS: Na_2_SO_4_; OF: OPC + FA (75:25); FA: fly ash; Tween 80: additive increasing the hydrophilicity of organic parts in the soil.Careful attention should be paid to the high imperfection of the above comparison. This is because the various tests used different amounts of binders in relation to the dry weight of the soil and different moisture contents of the mixtures, and different types of subsoil were treated. Furthermore, the contaminants contained in the soils were present in different concentrations. In addition to the aforementioned factors, the UCS is also affected by the shape and size of the samples, making the comparison subject to additional distortion. Nevertheless, in the present study, the authors have quoted the values obtained, due to the often quite different order of magnitude of the compressive strength achieved. The minimum value for the compressive strength of an S/S product is commonly considered as 0.350 MPa, which is the value specified by the US EPA for S/S waste in landfills [195]. For engineering purposes, it is necessary to know the design loads and stresses arising in the soil in order to estimate the minimum compressive strength of the S/S product constituting the subsoil for the proposed development.

## Data Availability

Not applicable.
